# Bacterial meningitis in children under 15 years of age in Nepal

**DOI:** 10.1186/s12887-015-0416-6

**Published:** 2015-08-19

**Authors:** Rajani Ghaju Shrestha, Sarmila Tandukar, Shamshul Ansari, Akriti Subedi, Anisha Shrestha, Rekha Poudel, Nabaraj Adhikari, Shital Raj Basnyat, Jeevan Bahadur Sherchand

**Affiliations:** Public Health Research Laboratory, Institute of Medicine, Maharajgunj, Kathmandu, Nepal; Department of Microbiology, Chitwan Medical College, Bharatpur, Chitwan, Nepal; Kantipur College of Medical Science, Sitapaila, Kathmandu, Nepal; Central Department of Microbiology, Tribhuvan University, Kirtipur, Kathmandu, Nepal

**Keywords:** Bacteria, Children, Meningitis, Nepal

## Abstract

**Background:**

Bacterial meningitis in children is a life-threatening problem resulting in severe morbidity and mortality. For the prompt initiation of antibacterial therapy, rapid and reliable diagnostic methods are of utmost importance. Therefore, this study was designed to find out the rate of bacterial pathogens of meningitis from suspected cases by performing conventional methods and latex agglutination.

**Methods:**

A descriptive type of study was carried out from May 2012 to April 2013. Cerebrospinal fluid (CSF) specimens from 252 suspected cases of meningitis were subjected for Gram staining, bacterial culture and latex agglutination test. The identification of growth of bacteria was done following standard microbiological methods recommended by American Society for Microbiology. Antibiotic sensitivity testing was done by modified Kirby-Bauer disk diffusion method.

**Results:**

From the total 252 suspected cases, 7.2 % bacterial meningitis was revealed by Gram staining and culture methods whereas latex agglutination method detected 5.6 %. Gram-negative organisms contributed the majority of the cases (72.2 %) with *Haemophilus influenzae* as the leading pathogen for meningitis. Overall, 33.3 % mortality rate was found.

**Conclusions:**

In conclusion, a significant rate of bacterial meningitis was found in this study prompting concern for national wide surveillance.

## Background

The presence of microorganisms in normally sterile body fluid specimens may be representative of life-threatening infections [1]. Infection of normally sterile body fluids often results in severe morbidity and mortality; therefore, rapid and accurate microbiological assessment of these specimens is important to successful patient management. Any microorganism found in a normally sterile site must be considered significant, and all isolates must be reported [2].

Bacterial meningitis, an infection of the membranes (meninges) and cerebrospinal fluid (CSF) surrounding the brain and spinal cord, is a major cause of death and disability worldwide [3]. In recent years, despite improvements in antimicrobial therapy and intensive care support, overall mortality rates related to bacterial meningitis of around 20 to 25 % have been reported by major centers [4, 5]. Early clinical suspicion and implementation of appropriate antimicrobial therapy are critical to minimize adverse outcomes [6–8]. Rapid diagnosis by Gram staining for a suspected infectious organism from a sterile body fluid is a major responsibility of clinical microbiology laboratory. In addition to suggesting a diagnosis, it guides clinicians in the choice of antibiotics [9–11]. Gram stain usually yields organisms, allowing presumptive diagnosis in the majority of cases with sensitivity of greater than 90 %, subsequently confirmed within 1 or 2 days by culture [12].

The current standard for the identification of bacterial meningitis in developing countries remains to be microscopic examination and consequent culture of CSF. However, this approach might have some disadvantages with regard to the desired rapidity and sensitivity [12], and the risk of false negative result is high because only a small number of microorganisms may be present in the specimens [13, 14]. An etiological diagnosis of bacterial meningitis may be possible in less than 1 hour by latex agglutination, co-agglutination, or counter-immuno-electrophoresis. These methods have sensitivity comparable to those of microscopy and culture [15–18], but are considerably less sensitive than Enzyme Linked Immunosorbent Assay (ELISA) or radio-immunoassay [19, 20]. Rapid diagnosis and empirical therapy of meningitis on the basis of clinical findings are important, because stable neurological sequel such as hearing loss, mental retardation, seizures, and behavioral changes may occur in up to one-half of survivors [21].

Therefore, the aim of this study were to document the bacterial causative agents in suspected meningitis cases by performing culture and rapid test, their antimicrobial susceptibility profile and to find out the mortality rate.

## Methods

The descriptive type of study was carried out in the Public Health Research Laboratory, Institute of Medicine, Tribhuvan University Teaching Hospital (TUTH), Maharajgunj, Kathmandu during one year period from May 2012 to April 2013. Being the leading institute of Nepal, TUTH provides the best care at tertiary level to every patient through integrated clinical services at an affordable cost to every community of Nepal.

### Study populations

A total of 252 CSF specimens, during one year study period, were collected from the patients of age up to 15 years having clinical features suggestive of meningitis.

### Collection and processing of CSF samples

After obtaining the consent, the CSF specimens were collected by medical officers in a sterile tube. The volume and gross appearance i.e., consistency, presence of blood and the color of CSF were noted. CSF specimen greater than 1 ml was centrifuged at the rate of 2,500 revolutions per minute (rpm) for 10 minutes to concentrate if any organisms were present. The sediment was used for culture as well as for Gram staining. The supernatant was saved for detection of antigen by latex agglutination commercial kit (BIO-RAD, Pastorex™ Meningitis). If CSF specimen less than 1 ml was received, the specimen was inoculated directly to the culture media.

### Bacteriological assays

All the specimens were collected from the patients of a single tertiary care hospital and thus, the specimens were transported to the laboratory within half an hour. CSF specimen was inoculated in MacConkey agar (MA), blood agar (BA) and chocolate agar (CA) plates. The MA and BA plates were incubated overnight at 37 °C aerobically and the CA plates were incubated up to 48 hours at 37 °C in 5 % CO_2_ atmosphere (i.e., in a CO_2_-incubator or a candle-jar) which improved growth of capnophilic bacteria. The bacterial growth obtained was examined for colonial as well as Gram staining characteristics and identification was done following standard microbiological methods recommended by American Society for Microbiology (ASM) [2]. A purity plate was employed to ensure that the inoculum used for the biochemical tests was pure. The bacterial growths were also subjected for the latex agglutination for grouping of bacterial strains according to the manufacturer instructions as described below.

#### Grouping of *Neisseria meningitidis* (A, B and C only), *Haemophilus influenzae, Escherichia coli* and *Streptococcus pneumoniae*

Bacterial suspension was prepared by taking 2–3 colonies of *Neisseria meningitidis* (*N. meningitidis*), *Haemophilus influenzae* (*H. influenzae*) and *Escherichia coli* (*E. coli*) and 10–12 colonies of *Streptococcus pneumoniae* (*S. pneumoniae*) and emulsifying in 30 microlitres (μl) of sterile normal saline on a disposable card. One drop of latex reagent was added to the bacterial suspension and mixed properly using a rod. The whole preparation was then rotated at 120 revolutions per minute (rpm) and observed for the appearance of any clear agglutination in less than 2 min. The identification of the species was confirmed using conventional biochemical tests.

#### Grouping of group B streptococcus (*Streptococcus agalactiae*)

Five colonies of group B streptococcus were suspended in 2 ml of Todd Hewitt broth and incubated for 2–3 hours at 37 °C in a water bath. Then, centrifuged for 5 min at 30,000 rpm. One drop of supernatant (40–50 μl) was placed on the disposable card and one drop of latex reagent was added on the drop of supernatant. The whole preparation was mixed with a rod and rotated at 120 rpm. It was observed for the appearance of any clear agglutination within less than 1 min. The identification of the species was confirmed using conventional biochemical tests.

### Antibiotic susceptibility test (AST)

All the isolates grown were subjected for antibiotic susceptibility testing by modified Kirby-Bauer disk diffusion method in compliance with Clinical and Laboratory Standards Institute (CLSI) guidelines using Mueller- Hinton agar standard media. The inhibition zone standards for antimicrobial susceptibility were considered from tables of interpretative zone diameters of CLSI [22]. Antibiotic disks (HiMedia Laboratories, Pvt. Limited, India) used were: ampicillin (10 μg), amoxicillin (10 μg), chloramphenicol (30 μg), ciprofloxacin (5 μg), rifampicin (15 μg), tetracycline (10 μg), imipenem (10 μg), co-trimoxazole (25 μg), vancomycin (30 μg). *Staphylococcus aureus* ATCC 25923 and *E. coli* 25922 were used as control organisms for antibiotic susceptibility testing.

### Latex agglutination test from the supernatant

Latex agglutination is an immunological technique based on the detection of soluble antigens released by the causative organisms into biological fluids like CSF during the infection by using latex particles coated with specific homologous antibodies, permits a more rapid diagnosis. The soluble antigens that can be detected are the polysachharides specific for certain serogroups or serotypes: *S. pneumoniae* (83 types), *H. influenzae* type b, *N. meningitidis* (group A, B, C and Y/W135), *E. coli* K1, and group B *streptococcus* (*Streptococcus agalactiae*). In the presence of homologous antibody, latex particles agglutinate, in the absence of antigen they remain in a homogenous suspension.

In the latex agglutination test, for the detection of antigens, one drop (40–50 μl) of the supernatant of CSF specimen was taken on agglutination card and one drop of each latex reagent was added. The specimen and the reagents were mixed using a rod (separate rod for each reagent). The whole preparations were then rotated at 120 rpm for 10 min. Any visible agglutination was noted and considered as positive.

### Ethical aspects

The verbal consent in local language for this study was taken from the guardians of participating children, then, written informed consent was obtained. This study was approved by the Institutional Review Committee (IRC) of Chitwan Medical College, Bharatpur, Chitwan, Nepal.

### Data analysis

All the results were entered in the worksheet of statistical package for social science (SPSS) software-16.0 version and the results were determined.

## Results

### Total and positive cases

During one year study period, 252 CSF samples were collected and processed for the detection of bacterial pathogens by culture and antigen detection method. The positive bacterial growth was obtained from 18 (7.2 %) cases and latex agglutination test for detection of antigen revealed 14 (5.6 %) positive cases (Table 1).

**Table 1:** Age-wise distribution of total and positive cases

Age group	Total suspected cases		Positive cases	
	No.	%	No.	%
<1 month	69	27.4	5	27.8
1–12 months	29	11.5	5	27.8
13–36 months	35	13.9	1	5.5
37–60 months	26	10.3	2	11.1
>60 months	93	36.9	5	27.8
Total	252	100	18	100

### Age-wise distribution of cases

The highest number of suspected cases were found in the age group of more than 60 months (36.9 %) followed by 27.4 % in age group of less than 1 month while the lowest (10.3 %) was found in the age group of 37–60 months. Likewise, the highest rate of culture positive cases were found in the age group of less than 1 month, 1–12 months and more than 60 months with 27.8 % in each age group while the lowest rate (5.5 %) was observed in age group of 13–36 months (Table 1).

### Gender-wise distribution of patients

Among total enrolled cases, in this study, 139 (55.2 %) were males and 113 (44.8 %) were females. Out of total 18 (7.2 %) positive cases, male and female showed equal distribution of culture positive cases that is 9 (3.6 %) (Table 2).

**Table 2:** Gender-wise distribution of total and positive cases

Sex	Total suspected cases		Positive cases	
	No.	%	No.	%
Male	139	55.2	9	3.6
Female	113	44.8	9	3.6
Total	252	100	18	7.2

### Comparison of detection methods

From 252 clinical specimens of CSF, examined for Gram staining, culture and latex agglutination test, Gram staining and culture detected 18 (7.1 %) positive cases whereas latex agglutination test detected 14 (5.6) specimens positive for bacterial antigen. All latex agglutination test positive cases showed bacterial growth in culture (Table 3).

**Table 3:** Comparison of different methods for the detection of positive cases

Method	No. of specimens	No. of positive cases (%)
Gram staining	252	18 (7.2)
Culture	252	18 (7.2)
Latex agglutination	252	14 (5.6)

### Distribution of bacterial isolates

Gram-positive as well as Gram-negative organisms were isolated from CSF specimens. Gram-negative aerobic rods accounted for 11 cases (61.1 %) and Gram-negative cocci occupied 2 cases (11.1 %) while Gram-positive cocci constituted the remaining 5 cases (27.8 %). The highest frequency and distribution was shown by *H. influenzae* (38.9 %) followed by group B *streptococcus* and *E. coli* each constituting 16.7 % while *Pseudomonas* spp. was found in 1 (5.5 %) case (Table 4).

**Table 4:** Age wise distribution of bacterial isolates

Organisms	<1 month	1–12 months	13–36 months	37–60 months	>60 months	Total
	No. (%)	No. (%)	No. (%)	No. (%)	No. (%)	No. (%)
Gram-positive	3 (60)	0	1 (20)	0	1 (20)	5 (27.8)
Group B *streptococcus*	1 (33.3)	0	1 (33.3)	0	1 (33.3)	3 (16.7)
*S. pneumoniae*	2 (100)	0	0	0	0	2 (11.1)
Gram-negative	2 (15.4)	5 (38.5)	0	2 (15.4)	4 (30.8)	13 (72.2 %)
*H. influenzae*	2 (28.6)	3 (42.8)	0	1 (14.3)	1 (14.3)	7 (38.9)
*N. meningitidis*	0	0	0	0	2 (100)	2 (11.1)
*E. coli*	0	2 (66.7)	0	0	1 (33.3)	3 (16.7)
*Pseudomonas* spp.	0	0	0	1 (100)	0	1 (5.5)
Total	5 (27.8)	5 (27.8)	1 (5.5)	2 (11.1)	5 (27.8)	18 (100)

### Antibiotic susceptibility pattern of isolates

Among the various antibiotics tested, tetracycline was found the most effective drug against Gram-positive as well as Gram-negative isolates. Resistance to tetracycline was only noted in *H. influenzae* (14.3 %). All of the *S. pneumoniae*, *E. coli* and *Pseudomonas* spp. were susceptible to chloramphenicol and ciprofloxacin while considerable rate of resistance (50 %) to ceftriaxone was only found in *S. pneumoniae* (Table 5).

**Table 5:** Antibiotic resistance rate of different isolates

Antibiotics	*S. pneumoniae* (2)	Group B streptococcus (3)	*H. influenzae* (7)	*N. meningitidis* (2)	*E. coli* (3)	*Pseudomonas* spp. (1)
	No. (%)	No. (%)	No. (%)	No. (%)	No. (%)	No. (%)
Ampicillin	2 (100)	-	2 (28.6)	2 (100)	-	-
Ceftriaxone	1 (50)	0	1 (14.3)	0	0	0
Chloramphenicol	0	1 (33.3)	1 (14.3)	1 (50)	0	0
Ciprofloxacin	0	1 (33.3)	3 (42.9)	0	0	0
Rifampicin	-	-	2 (28.6)	0	-	-
Amoxicillin	-	-	1 (14.3)	-	-	-
Tetracycline	0	0	1 (14.3)	-	0	0
Imipenam	-	-	-	0	1 (33.3)	0
Cotrimoxazole	2 (100)	2 (66.7)	3 (42.9)	2 (100)	2 (66.7)	1 (100)
Vancomycin	0	1 (33.3)	-	-	-	-

### Mortality rate

Among total positive cases, mortality rate was found to be 33.3 %. Half (50 %) of the cases infected with *N. meningitidis* and *S. pneumoniae* were died followed by *H. influenzae* (42.9 %) and *E. coli* (33.3 %) (Table 6).

**Table 6:** Mortality rate among culture positive cases

Organisms	Total positive cases	Live	Death
	No.	No. (%)	No. (%)
*H. influenzae*	7	4 (57.1)	3 (42.9)
*N. meningitidis*	2	1 (50.0)	1 (50.0)
*S. pneumoniae*	2	1 (50.0)	1 (50.0)
Group B streptococcus	3	3 (100)	0
*E. coli*	3	2 (66.7)	1 (33.3)
*Pseudomonas* spp.	1	1 (100)	0
Total	18	12 (66.7)	6 (33.3)

## Discussion

The meningitis can be caused by bacterial or viral pathogens but the viral meningitis is usually less severe and its frequency increases slightly in the summer months because of greater exposure to viruses [23]. Despite advances in management, bacterial meningitis remains a life threatening infection with high rates of morbidity and mortality [24]. Early clinical suspicion and implementation of appropriate antimicrobial therapy are critical to minimize adverse outcomes. CSF culture is considered the diagnostic reference standard for bacterial meningitis, and bacterial isolation is important for antimicrobial susceptibility testing and molecular epidemiology [6–8]. Molecular techniques are quickly becoming the gold standard for both bacterial and viral meningitis but these methods are not easily available in Nepal because of their high cost. Therefore, standard used for our comparisons was a positive result by any of the three test methods i.e. by Gram’s staining method, CSF culture and latex agglutination. Of the 252 CSF samples, only 18 (7.2 %) revealed significant growth in this study. In similar studies conducted in Nepal, significant growths of various isolates were also detected by Shah et al. (3.7 %) in 2001 [25] and Ansari et al. (4.4 %) in 2011 [26]. Proportionately, a high rate of bacterial growth (17.7 %) was detected by Wu et al. in 2013 [27]. The rate of positive yield of bacterial meningitis depends on a number of factors for examples, the time of lumber puncture, the probable number of bacteria in Gram’s stained preparation of CSF and the antibacterial therapy prior to lumberpuncture [28].

In this study, among the enrolled cases, up to 12 months of age was found to be the most vulnerable age group in children accounting for 55.6 % of total positive cases. Similarly, the higher rate of meningitis (32.1 %) was also detected in children of 6–12 months of age by Joshi Batajoo from Nepal [29]. The higher rate of meningitis in early age children may be because of the underdeveloped immune system.

Bacteriological profile (Gram staining and culture result) of CSF facilitates the choice of antibiotic therapy [30]. CSF culture sensitivities typically range between 70 to 90 % [6–8] with variation in case inclusion criteria, patient characteristics, laboratory practices, and spectrum of bacterial pathogens likely contributing to the observed differences [27]. Gram staining, a mainstay of bacterial meningitis diagnosis, is widely available, inexpensive and rapid [24]. Some authors recommend using latex agglutination tests to detect bacterial capsular antigens in patients with suspected bacterial meningitis who have been receiving antibiotics at the time the lumbar puncture is performed. These tests are not specific, however, and they identify very few cases of bacterial meningitis not already detected by CSF culture [31]. In our study, the latex agglutination test showed sensitivity of 77.8 % when compared with Gram staining and bacterial culture. This may be due to that currently available latex agglutination antigen detection test kits have several limitations [32–34]. Despite it is simple to perform, does not require special equipment, and is rapid (results are available in ≤15 min). Depending on the meningeal pathogen, latex agglutination has shown good sensitivity in detecting the antigens of common meningeal pathogens: 78-100 % for *H. influenzae* type b, 67–100 % for *S. pneumoniae*, 69–100 % for *S. agalactiae*, and 50–93 % for *N. meningitidis*. However, a negative bacterial antigen test result does not rule out infection caused by a specific meningeal pathogen [35].

Etiology of bacterial meningitis varies from place to place [36, 37]. Of the 252 samples being only 7.2 % culture positivity, Gram-positive bacteria was isolated from 5 (27.8 %) of culture positive cases and Gram-negative from 13 (72.2 %) cases which was in accordance with the study conducted by Ghotaslou et al. from Iran in 2012 [38].

In infants and young children worldwide, *S. pneumoniae* is the common Gram-positive agent for bacterial meningitis whereas *N. meningitidis* and *H. influenzae* type b (Hib) are the most common Gram-negative causes of bacterial meningitis. Among children older than 5 years of age and adolescents, *S. pneumoniae* and *N. meningitidis* are the predominant causes of bacterial meningitis [39, 40]. Immunization is the most effective means of prevention of bacterial meningitis in children. Universal immunization with the conjugated vaccines has been associated with a reduction of more than 99 % in invasive diseases caused by *H. influenzae* type b in developed countries [41]. The costs and implementation of these vaccines in developing countries pose daunting problems. The Child Health Division (CHD) of the Department of Health Services (DOHS) of Nepal introduced its regular immunization program by November of 2014 to prevent pneumonia and meningitis caused by 23 pneumococcal strains out of more than 80 strains. But it was not possible to cover the whole nation at once [42]. Among culture positive organisms, *H. influenzae* (38.9 %) was reported as a leading cause of meningitis followed by group B *streptococcus* (16.1 %) and *E. coli* (16.1 %) in this study. Out of 7 *H. influenzae* positive cases, 5 (71.4 %) was detected in children below 12 months. Despite of the *H. influenzae* type b (Hib) vaccination, it has been found as a leading cause of meningitis. The vaccination coverage and *H. influenzae* capsular types other than b could be the reason. However, the increased rate in some ethnic groups and some families, and the observation that siblings of patients with meningitis can have deficient antibody synthesis against *H. influenzae*, indicate that genetic predisposition to infection probably exists [43]. The risk of acquiring a secondary case of haemophilus or meningococcal disease is greatly increased after exposure to primary infection in the household [44]. Although, group B streptococcus is a leading cause of meningitis in neonates, this organism has been recognized with increasing frequency as a substantial cause of meningitis in later age groups [45]. In this study, we also identified a single case of meningitis with group B streptococcus in later age group. It has been defined that the serious underlying diseases could be the reason but in this study we could not evaluate the predisposing factors for the meningitis by group B streptococcus.

Once the bacterial meningitis is suspected, there should be no delay in starting appropriate empiric antibiotics. This requires knowledge of the likely pathogens and their antibiotic susceptibilities. In this study, the antibiotics like tetracycline, chloramphenicol and ciprofloxacin were found most effective against Gram-positive as well as Gram-negative whereas, vancomycin was most effective against Gram-positive while ceftriaxone was the most effective against Gram-negative isolates. The result of our study corroborates with Mastro et al. (1991) who reported only 2.8 % of strains resistance to chloramphenicol [46]. Ampicillin and cotrimoxazole were the least effective antibiotics against the isolates. All *S. pneumoniae* and *N. meningitidis* isolates were resistant to ampicillin and cotrimoxazole in our study. By contrast, higher resistance rate (64.1 %) to cotrimoxazole was also reported by Saha et al. in 1997 from Nepal [47]. The increased rate of cotrimoxazole resistance can be possibly be correlated with the wide use of this antibiotic in the communities because of its dose convenience, cost effectiveness, easy availability and the practice of prescribing it to treat suspected bacterial pneumonia cases.

Out of 18 infected children, 6 (33.3 %) children died from the bacterial meningitis. Case-fatality rate was most common in meningitis caused by *Neisseria* spp. (50.0 %) and *S. pneumoniae* (50.0 %) followed by *H. influenzae* (42.9 %) and *E. coli* (33.3 %) in this study. The mortality rate of 4 to 10 % due to bacterial meningitis in children has been revealed in more recent studies [48] however, a similar high mortality rate of 48 % was also detected in a study by Al-Harthi et al. from Saudi Arabia [49].

A differential diagnosis of meningitis requires the differentiation of viral, tuberculous and acute bacterial meningitis and prevention from transmission of diseases, vaccination against the organism, their chemoprophylaxis are suggested in order to prevent the disease from being transmitted as well as a cause for mortality of children.

## Conclusion

The bacterial meningitis is abundant in children up to 15 years of age as revealed in the present study. A variety of Gram positive as well as Gram negative organisms was documented. This situation calls for the longitudinal nation-wide surveillance for bacterial meningitis to document the real scenario regarding prevalent strains of bacterial pathogens in order to implement or change the vaccine types. We also recommend health education of the public to improve awareness on adequate immunization and appropriate drug management.
